# Large-Group One-Session Treatment: A Feasibility Study of Exposure Combined With Applied Tension or Diaphragmatic Breathing in Highly Blood-Injury-Injection Fearful Individuals

**DOI:** 10.3389/fpsyg.2018.01534

**Published:** 2018-08-21

**Authors:** André Wannemueller, Alessa Fasbender, Zarah Kampmann, Kristin Weiser, Svenja Schaumburg, Julia Velten, Jürgen Margraf

**Affiliations:** Mental Health Research and Treatment Center, Ruhr University Bochum, Bochum, Germany

**Keywords:** exposure treatment, group treatment, one-session treatment, large-group one-session treatment, blood-injury-injection phobia

## Abstract

**Objective:** Large-group one-session treatments (LG-OSTs) might represent a promising treatment tool as increasing evidence suggests their effectiveness in individuals with different situational fears. In the present study, we explored feasibility and effectiveness of an exposure-based LG-OST protocol applying applied tension and diaphragmatic breathing as coping strategies in a sample of 40 individuals, highly fearful of blood-injury-injection (BII).

**Method:** We assessed participants’ BII-fear using questionnaires and a behavioral approach test (BAT) before and after treatment, consisting of a blood-drawing procedure. Stability of treatment effects was assessed via online-survey at 7-month follow-up.

**Results:** The LG-OST procedure evidenced feasible and effective. Pre-post treatment comparisons showed medium to large treatment effects (*d* = 0.40–0.93) regarding the questionnaire measures. After being treated, 70% of the individuals successfully underwent a blood drawing. Moreover, participants continued to improve in the post follow-up interval leading to large treatment effects (*d* = 1.19–1.62).

**Conclusion:** In treating BII-fear, LG-OSTs might not only serve within a framework of a stepped care approach but also could represent a useful single-treatment option. Additionally, due to their high efficiency and standardization of treatment delivery, LG-OST protocols might foster research at the interface of basic and clinical research.

## Introduction

With a reported 12-month prevalence rate between 7 and 9% (Wittchen et al., 2011), Specific Phobias (SPs) are among the most frequent single mental disorders in adults in Western countries. Although the disability levels vary between SPs and may also include relatively minor impairments (e.g., in spider phobia or snake phobia, at least for individuals living in industrialized countries), most SPs (e.g., dental phobia, emetophobia) cause affected individuals great distress and significant functional impairment (e.g., Essau et al., 1999). Moreover, considering the early onset of SPs, its high comorbidity and persistence (Wardenaar et al., 2017) as well as its common harmful consequences, such as more annual work loss days associated with SPs than chronic heart- or lung disease (Alonso et al., 2004), the economic relevance of SPs is substantial.

With a point prevalence ranging between 2 and 3% in European countries (Becker et al., 2007; Depla et al., 2008), the subtype of blood-injury-injection (BII) phobia is quite frequent. Although just like in other phobia-subtypes, a main feature of BII-phobia is an intense, persistent subjective fear response difficult to control when phobia-related stimuli are present or anticipated (see American Psychiatric Association [APA], 2013 for all symptom criteria) BII-phobia is distinctive among SPs due to its unique physiological response pattern differing from other phobia subtypes. In about 75% of cases, an initial sympathetic activating response is followed by a vasovagal response (American Psychiatric Association [APA], 2013). Physiologically, this diphasic pattern first described by Graham et al. (1961) leads to pre-syncopal symptoms (e.g., nausea, blurred vision) and may result in fainting and loss of consciousness triggered by a decreased transient cervical perfusion. A sympathetic fear activation conflicting with a parasympathetic disgust response has been hypothesized as a possible explanation for this pattern (e.g., Page, 2003). Indeed, individuals with BII-phobia often report high levels of disgust when exposed to phobia-relevant stimuli, and reactivity to disgust elicitors has been identified as a risk factor for the development of BII-phobia (e.g., de Jong and Merckelbach, 1998; Olatunji et al., 2008). Fainting can also be a central factor in the etiology of BII-fear, where classical conditioning is proposed as one of the most prominent explanations for its development (e.g., Öst, 1991). The BII-subtype is associated with avoidance of essential health care interventions and with an enhanced risk of severe health impairments at older age (Miloyan and Eaton, 2016). Moreover, BII-phobia is amongst those SPs individuals most frequently seek professional treatment for (Agras et al., 1969). Therefore, it can be considered to be among the most debilitating SPs.

The cost of health care as well as the impact on the individual in terms of suffering and disability call for the development of treatment approaches and prevention strategies which are not only highly effective but also highly efficient and, especially in the case of SPs, lower the threshold of treatment access. Efficiency is an important aspect due to the limited number and capacity of clinical professionals. Improving access to treatment is vital, since many individuals suffering from phobia-like symptoms are uncertain whether their problems justify professional treatment, whereby denial may lead to unnecessary chronification of symptoms.

In terms of duration, one-session treatments (OSTs) can be seen as highly efficient treatment approaches in the field of SPs. Moreover, compared to multi-session treatments, treatment delivery in just one session may ensure that patients receive the intended dose of treatment. OSTs, first introduced by Öst (1989), last for about 3 h or less and were originally designed to combine elements of *in vivo* exposure with participant modeling. However, cognitive and motivational aspects have been added through psychoeducative elements, skills training, reinforcement, and cognitive challenges (see Zlomke and Davis, 2008 for a review). So far, OSTs have been applied in a wide range of phobic disorders, such as spider phobia (Öst et al., 1991b; Hellström and Öst, 1995), flight phobia (Öst et al., 1997a), dental phobia (Haukebø et al., 2008), and agoraphobia (Öst et al., 2001) and were demonstrated to reduce SP-symptoms very effectively. Concerning long-term outcome, however, treatments provided in multi-session formats were shown to slightly outperform the effects of OSTs (for a review of findings see Wolitzky-Taylor et al., 2008).

Due to the aforementioned distinct physiological responding, CBT-based treatments targeting BII-fear often combine exposure strategies with applied tension, which consists of alternating tension and release of large skeletal muscles to counteract bradycardia and hypotension (see Kozak and Montgomery (1981) and Krediet et al. (2002) for more detail). A meta-analysis (Ayala et al., 2009) concerning treatment strategies in the field of BII concluded that all treatment strategies applied in these studies, that is, pure exposure (Öst et al., 1984, 1991a, 1992), applied tension (Öst et al., 1989, 1991a; Hellström et al., 1996), tension only (Öst et al., 1991a; Hellström et al., 1996) applied relaxation (Öst et al., 1984, 1989) as well as a combination of applied tension and applied relaxation (Öst et al., 1989) led to significant clinical improvement at post-treatment, with exposure slightly outperforming all other techniques (Ayala et al., 2009). Treatments proved effective, also when administered in a one-session format (Öst et al., 1992). A recent study (Meuret et al., 2017) demonstrated that even very brief (12 min) video-based instructions of applied tension or a hypoventilation breathing techniques led to substantial reductions of subjective and bodily-symptoms during exposure to BII-relevant stimuli.

Besides using one-session formats, treatment efficiency may greatly improve by reducing the therapist-patient ratio, which is the case in a group setting. So far, three studies delivered OSTs in small group settings (Öst, 1996; Öst et al., 1997b; Götestam, 2002). All three studies targeted spider phobia and reported substantial fear reduction resulting from the one-session small group approach. Öst (1996) compared differential treatment effects in relation to group size and did not observe significant differences regarding the efficacy resulting from smaller groups (*n* = 3–4) compared to larger groups (*n* = 7–8) in most measures. However, the author reported a trend for better effects for the small group condition. In sum, existing studies on small group OSTs proved feasible and effective, especially when employing direct rather than indirect exposure strategies.

Encouraged by the positive reports of small group OSTs, Wannemueller et al. (2016, 2017) recently conducted two Phase I open trials applying one-session formats in large-group settings (LG-OSTs). LG-OST proved feasible in a sample of *N* = 78 spider fearful individuals as well as in a sample of *N* = 43 dental fearful individuals. Moreover, both treatments led to substantial short-term as well as long-term reductions of subjective fear, and spider fearful individuals showed less behavioral avoidance after LG-OST. However, LG-OST was useful for some but not all patients; effectiveness in dental-fear participants was lower compared to spider-fear participants, and LG-OST effects altogether were lower compared to those reported for individual treatment formats.

Furthermore, LG-OST protocols might be useful instruments to investigate possible mediators and moderators of treatment outcome. So far, drawing conclusions on those factors is difficult, because often quite unstandardized treatment formats are applied in heterogeneous samples. LG-OST protocols might enable the identification of treatment moderators under highly standardized treatment conditions. Moreover, due to their high efficiency with respect to recruitment-, cost-, and time-related aspects, exposure-based LG-OSTs could enable an easy and direct transfer and testing of mechanistic lab-based findings on fear extinction in clinical contexts.

Treating situational fears in exposure-based one-session large group designs might contribute to both high treatment efficiency and low-threshold treatment access. Moreover, high treatment standardization in LG-OSTs might foster research at the cross-roads of basic and applied clinical research. Therefore, we developed a LG-OST protocol containing well-evaluated strategies for BII-phobia. In the Phase I study, we investigated the feasibility and effectiveness of the LG-OST protocol in a group of 40 participants and explored potential outcome-predictors of LG-OST. We hypothesized that the LG-OST would lead to a substantial reduction in BII-fear, analogous to the results observed in the spider- and dental-fear cohort (Wannemueller et al., 2016, 2017). Prior to treatment, we assessed trait anxiety as a potential outcome predictor, as trait anxiety has been identified to influence treatment outcome (e.g., Muris et al., 1998).

## Materials and Methods

### Participants

Participants for the Phase I study were recruited by advertisements on local radio, newspapers, and social networks and could register online for participation on a website established for the project. The website provided detailed information about time, location, and the general structure of the LG-OST program. However, we refrained from providing detailed information concerning the treatment contents in order not to deter anyone interested from participating. There were only two inclusion criteria: high subjective fear of blood and/or injuries and/or injections; avoidance of relevant situations such as blood drawings and being of full age. Prior to registration, participants could screen their level of BII-fear and check if they were eligible for participation by completing an online-version of the short-version of the Multidimensional Blood/Injury Phobia Inventory (MBPI-K, Gebhardt et al., 2010, see the measures section for a detailed description). We recommended participation in LG-OST if the total score exceeded 32, representing a score higher than the mean plus one standard deviation of the standardization sample of the MBPI. However, we did not apply fixed cut-off scores, which in case of falling short would have led to exclusion.

**FIGURE 1:**
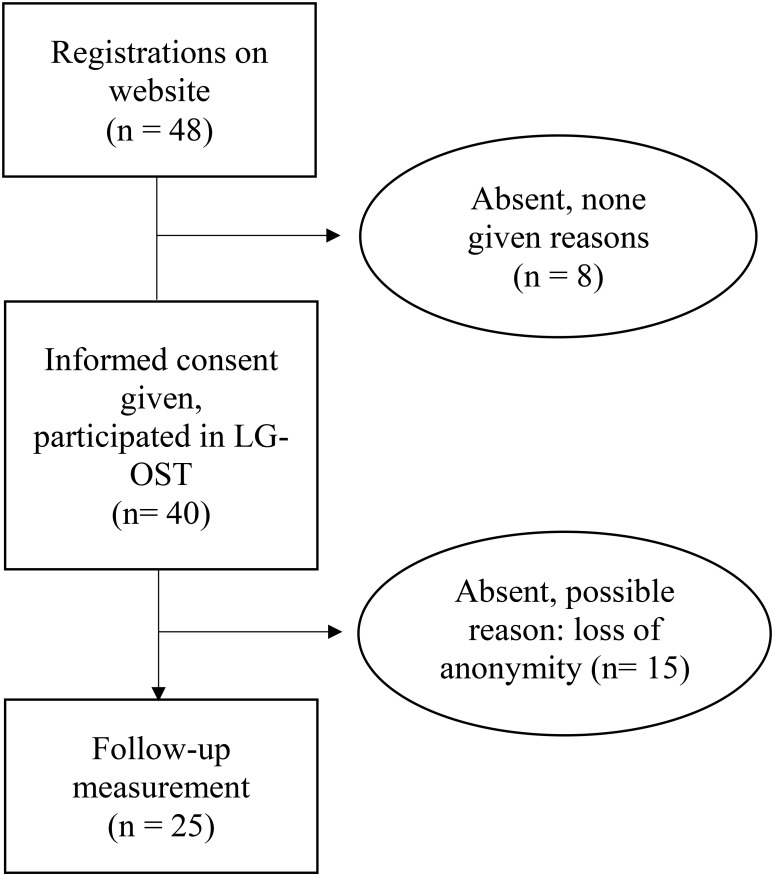
Flow diagram of patients entering the study.

Initially *N* = 48 individuals registered online for participation. Finally, on the day of the group treatment, *N* = 40 participants, all Caucasian (34 female, 6 male) with a mean age of 26.63 (*SD* = 8.21) years appeared at the treatment-center where they gave their informed consent to attend LG-OST, see **Figure 1**. The local Ethics Committee of the psychological faculty where the study was conducted, approved the study. This feasibility study was a non-registered trial.

### Procedure

Potential participants were informed on our website that they would remain anonymous during the treatment. A well-trained licensed clinical psychologist experienced in treating SPs performed the LG-OST. It was preceded and followed instantly by a behavioral approach test (BAT). During the BAT, a blood drawing procedure was carried out with the participants. Moreover, they completed a set of questionnaires, assessing subjective components of blood, injury, mutilation, and injection fear pre- and post-intervention, i.e., at the same day after the treatment. Before leaving, all participants were asked to voluntarily sign-up and to be available for follow-up (FU)-measures. Twenty-five participants (62.5%) registered. After 7 months (*M* = 7.4; *SD* = 2.96), we invited them for an online FU-assessment consisting of the same questionnaires.

### Large-Group One-Session Treatment (LG-OST)

The LG-OST was delivered to the patients in an auditorium of the university where the study was conducted and lasted for about 160 min. It consisted of three phases: a psychoeducation phase, a training phase and an exposure phase.

#### Psychoeducation Phase (About 40 min)

After entering the auditorium, participants received information about a typical medical blood drawing- and vaccination procedure by a 20-min movie-clip. In this clip, a medical specialist explained the procedure typically used for blood-drawings and vaccinations and showed the materials used (e.g., tourniquet, butterfly needle, disinfectants). He targeted frequently asked questions, such as the probability of occurrence of unwelcome side effects, for example hematoma or infections.

Afterward, a psychotherapist explained the nature and utility of fear and its cognitive, behavioral and subjective consequences, according to the three-level approach of fear (Lang, 1979). He outlined the bodily responses of BII-fear being unique since the initial arousal response, typically occurring during fear responses is often followed by an exaggerated vasovagal counter-regulation. Afterward, he introduced some common sympathetic (e.g., palpitation, sweating) and vasovagal (e.g., dizziness, nausea) symptoms and explained that the latter might culminate in a vasovagal syncope if not counteracted. By reference to an animated picture slide, participants learned to classify their individual symptoms into vasovagal or sympathetic categories. Afterward, they were taught the rationale of applied tension and diaphragmatic breathing. The therapist pointed out that vasovagal fear responses could be overcome by the use of strategies preventing the decrease of blood-pressure during exposure to individual fear cues. He further explained that the use of a relaxed breathing technique could markedly decrease sympathetic activity and reduce stress and tension (Busch et al., 2012).

#### Training Phase (About 40 min)

The training phase was divided into two parts: training of diaphragmatic breathing and training of applied tension. The techniques were instructed by a psychotherapist with clinical experience in the treatment of SPs. During the training of diaphragmatic breathing (about 20 min) participants were instructed to concentrate on deep, calm exhalation. The therapist encouraged participants to apply the deep breathing technique during the following exposure exercises in order to deal with upcoming fear responses. The exercise was the same as described in Wannemueller et al. (2017). The concept of applied tension training (about 20 min) was based on the suggestions introduced by Öst and Sterner (1987) and modified for the use of brief training-intervals as proposed by Hellström et al. (1996). The exercise was instructed and demonstrated by a therapist. In a first step, patients were instructed to tense their leg- and gluteal muscles for a 15–20 s interval. After a 20 s recovery phase, they were asked to additionally tense their arm and hand muscles. Participants were encouraged to apply applied tension in order to respond to any upcoming vasovagal fear symptoms.

#### Exposure Phase (About 80 min)

Exposure consisted of three phases: Exposure with phobia relevant pictures, video-exposure, and live exposure. During pictorial exposure, 14 BII-relevant pictures stemming from an open access picture gallery depicting injuries, blood-drawing procedures, and injections were presented for 60 s each. Prior to the intervention, we piloted the pictures. A pool of 33 pictures, initially selected with respect to relevance, was rated by 20 independent raters concerning arousal (0–10) and valence (-5–5). Pictures generating the highest total scores were finally selected for presentation. After exposure to the pictures, participants watched a video-clip showing the psychotherapist undergoing a blood drawing procedure. During the whole process, he talked about his sensations aiming to provide a transparent insight into his thoughts and feelings for the viewers. Subsequent live-exposure consisted of a live blood drawing with one volunteer from the auditorium while the others observed the scene (for the volunteer live blood drawing served as the post-treatment BAT). He was encouraged to describe his impressions, thoughts and bodily sensations and to counteract any upcoming fear response with diaphragmatic breathing or applied tension, respectively. Blood was drawn by a medical professional.

The authors willingly provide the applied exposure materials on enquiry.

### Measures

#### Behavioral Approach Test (BAT)

A BAT was performed at pre- and post-treatment assessment. It was introduced by the therapist. Participants were instructed to undergo eight steps: 1. To enter the treatment room where the blood drawing would take place 2. To take a seat on a chair and uncover an arm. 3. To permit a medical professional to apply a standard tourniquet and stow a vein above the anticipated puncture site near the crook of the arm. 4. To permit the medical professional to place a wrapped cannula and a blood collection tube next to the patient. 5. To permit the medical professional to unpack the needle. 6. To permit the medical professional to place the needle in position to the anticipated puncture site. 7. To permit the medical professional to take a blood sample. 8. To closely look at the blood running into the cannula. Due to ethical reasons, we were not allowed to conduct the blood-drawing procedure two times on the same day. Therefore, the 7th and 8th step of the BAT could only be conducted at post-treatment assessment. Hence, the 6th step was the maximum step reachable at pre-assessment. However, participants were not explicitly informed that the procedure was stopped after step 6 at the pre-treatment BAT. During each step of the BAT, participants were asked to rate their subjective distress (SUD) and disgust intensity on an 11-point scale (0–10).

#### Subjective Blood-Injury-Injection Fear Measures

The German *Blut- Verletzungs- Spritzenangst Fragebogen* (*BVSF*, engl. Transl. “*Blood-Injury- Injection Fear Questionnaire,”* Voßbeck-Elsebusch et al., 2012) consists of 20 items assessing subjective BII-fear. Participants are asked to rate their subjective degree of “worry” in a specific BII-relevant situation (1 = not at all/7 = extremely). Hence, the total score of the BVSF ranges from 20 to 140. Factorial item-analysis yielded a 4-factor solution (injections; medical emergencies, and mutilations; blood and injuries in oneself; blood and injuries in others). According to the authors, they can be treated as separate subscales. The authors of the BVSF report good reliability (*r*_tt_ = 0.78) and validity indices. We found an internal consistency of Cronbach’s α = 0.91 in our sample. Sensitivity analyses of the BVSF yielded a cut-off score of 67 to reliably indicate the presence of a BII-phobia.

The German version of the *Mutilation Questionnaire (MQ*, Klorman et al., 1974) mainly assesses psychophysiological indices in and avoidance of BII-relevant situations with a main focus on wounds and mutilations. It consists of 30 dichotomous items (yes/no). The authors reported mean scores of *M* = 10.48 (*SD* = 5.90) in women and *M* = 7.49 (*SD* = 4.92) in men. For the MQ good reliability indices (Cronbach’s α) with *r* = 0.83 and *r* = 0.86 have been reported (Kleinknecht and Thorndike, 1990). In our sample we found an internal consistency of Cronbach’s α = 0.73.

The German version of the *Multidimensional Blood/Injury Phobia Inventory* (*MBPI*, Gebhardt et al., 2010) consists of 40 items measuring cognitive, behavioral, and emotional aspects of BII-fear. Participants are asked to rate whether they experience a respective symptom in a BII-relevant situation (0 = never/4 = always). The items constitute six subscales (injections; hospitals; fainting; own blood; own injury; blood/injuries in others). The cut-off score of 48 identified the existence of a BII-phobia with a sensitivity of 1.0 and a specificity of 0.96. The authors report a very high reliability of the total scale (Cronbach’s α = 0.94) and good reliability indices for the subscales (all Cronbach’s α >0.80). In our sample, internal consistency of the MBPI total scale was comparably high (Cronbach’s α = 0.95).

#### Clinical State Measures

Clinical state measures were used in order to identify potential predictors for treatment outcome. The German version of the *State-Trait Anxiety Inventory* (*STAI*, Laux et al., 1981) consists of two subscales, each describing emotional states in 20 statements at present (state scale) and during the last 2 weeks (trait version). Scores range from 20 (no anxiety) to 80 (high anxiety). Whereas the state-scale is highly sensitive for change, the trait-scale has a high retest reliability (*r*_tt_ = 0.96). In our sample, the internal consistency was Cronbach’s α = 0.90 for the trait-scale and α = 0.95 for the state-scale.

We used the German version of the *Beck Depression Inventory* (*BDI*, Hautzinger et al., 1994) to assess levels of depressiveness in our sample. It consists of 21 items measuring the existence and degree (0–3) of depressive symptoms within the last week. Score levels between 11 and 17 indicate a mild depressive symptomatology. Scores >17 can be considered as clinically relevant. In a German sample, the authors report an internal consistency of Cronbach’s α = 0.88. In our sample internal consistency of the BDI was Cronbach’s α = 0.79.

#### Disgust Sensitivity

In order to assess disgust sensitivity, we used the German “*Fragebogen zur Erfassung der Ekelempfindlichkeit”* (*FEE*, engl. Transl. “*Disgust Sensivity Questionnaire,”* Schienle et al., 2002). It consists of 37 5-stepped items aimed to assess disgust sensitivity in five basic disgust-categories (Death: *“You touch a dead body”;* Excrements: “*You notice an unpleasant smell and detect that you have stepped into dog dirt”;* Degenerated matter: *“You are about to drink a glass of milk when you smell that the milk is spoiled”;* Hygiene: *“On a bus a person sits next to you with an intense body odor”;* Oral defense: *“You smell vomit.”).* The authors reported high internal consistency of the FEE (Cronbach’s α = 0.90) and positive correlations between the FEE total score and BII-fear (*r* = 0.47) as well as between FEE-score and compulsivity (*r* = 0.25). In our sample internal consistency of the FEE was Cronbach’s α = 0.94.

#### Subjective Rating of Therapy Success

We used a 7-item Likert-scale to assess subjective treatment success. Participants were asked to rate subjective state changes from 1 (*much worse*) to 7 (*much better*). A score of four indicates no subjective change.

### Statistical Analysis

Because BII-fear related measures were likely to be correlated, analysis on pre-post change was carried out using a repeated measures MANOVA containing six measures, that is, BVSF, MBPI, MQ (sum scores), and BAT, including both SUD ratings (fear and disgust). There was no abrupt discontinuation during treatment, therefore, the analysis could be conducted as a completer analysis. Between measures, *n* could slightly differ due to falsely completed questionnaires. Therefore, we additionally report the results of single *post hoc* repeated measures ANOVAs conducted for each measure separately containing the exact *n* for the respective test. We calculated within-group effect sizes using Cohen’s *d* formula based on pooled standard deviations (Cohen, 1988).

Of the participants 62.5% (*n* = 25) were available for FU-assessment. In order to test whether drop-out was selective, we initially compared FU-completers and non-completers with univariate ANOVAs for each pre-treatment and all outcome measures. Stability of LG-OST effects was analyzed using a 3 × 5 repeated measures MANOVA within FU-completers. Treatment outcomes (pre- to post-difference scores) of all BII-related questionnaires and the BAT were correlated with pre-treatment fear levels, sociodemographic and trait-variables using Pearson-correlation analyses to explore possible predictors of the LG-OST outcome.

## Results

### Is LG-OST Effective in Reducing BII-Fear?

Repeated measures MANOVA yielded a highly significant effect, *F*(6,34) = 15.65, *p* < 0.001. For *post hoc* repeated measures separate ANOVAs including the BVSF, MBPI, BAT (steps), and BAT (SUD-fear and disgust), see **Table 1**.

**Table 1:** Means, SDs, and effect strengths (Cohen’s d) of pre- to post-changes in measures assessing blood-injury-injection fear in LG-OST-participants.

	LG-OST (n = 40)			ES^a^ [95% CI]
	Pre	Post	Pre vs. Post	
	*M (SD)*	*M (SD)*	*F*	*Cohen’s d*
***Sample characteristics, clinical data, and disgust sensitivity***			
Age (years)	26.63 (8.21)	–	–	–
Education (years)	14.33 (2.84)			
STAI-S	48.15 (13.08)	40.15 (13.03)	18.18***	0.61 [0.16 to -1.06]
STAI-T	41.28 (9.33)	–	–	–
BDI	8.21 (5.62)	–	–	–
FEE (tot)	84.68 (25.53)	–	–	–
*Death*	13.93 (6.50)	–	–	–
*Excrements*	16.35 (5.87)	–	–	–
*Deg. material*	18.25 (6.08)	–	–	–
*Hygiene*	20.23 (7.53)	–	–	–
*Oral defense*	15.93 (5.14)	–	–	–
***BII-fear measures***				
BVSF (tot)	87.20 (23.17)	66.23 (22.08)	48.76***	0.93 [0.47 to 1.39]
*BI-self*	19.90 (8.40)	15.93 (7.74)	26.14***	0.49 [0.05 to 0.94]
*BI-others*	19.95 (6.99)	14.98 (6.26)	44.13***	0.75 [0.30 to 1.20]
*Injection*	23.05 (3.80)	16.95 (5.60)	60.62***	1.28 [0.79 to 1.76]
*Mutilation*	24.30 (9.66)	18.38 (8.48)	28.22***	0.65 [0.20 to 1.10]
MQ (tot)	18.83 (5.40)	16.58 (5.84)	13.31**	0.40 [-0.04 to 0.84]
MBPI (tot)	85.83 (32.06)	60.48 (34.04)	54.61***	0.77 [0.31 to 1.22]
*Injection*	24.55 (7.20)	17.28 (8.81)	44.30***	0.90 [0.44 to 1.36]
*Fainting*	11.05 (7.98)	8.45 (7.90)	22.35***	0.33 [-0.11 to 0.80]
*Blood others*	19.13 (9.77)	13.08 (9.21)	31.87***	0.64 [0.19 to 1.09]
*Hospitals*	10.56 (9.16)	7.08 (7.89)	26.91***	0.41 [-0.04 to 0.85]
*Blood self*	8.44 (5.28)	6.00 (5.07)	21.16***	0.47 [0.03 to 0.92]
*Injuries*	9.90 (4.25)	7.15 (4.24)	40.75***	0.65 [0.20 to 1.10]
**BAT**				
Steps (max^b^)	5.28 (1.47)	5.73 (1.01)	4.79*	0.36 [-0.09 to 0.79]
Steps (max^c^)	5.28 (1.47)	6.88 (1.56)	46.65***	1.06 [0.59 to 1.52]
SUD^d^ fear 0-100	74.65 (23.94)	59.14 (29.93)	12.28**	0.57 [0.1938 to 1.02]
SUD^d^ disg. 0-100	29.31 (32.51)	23.21 (25.85)	3.08	–
GSR	–	5.65 (1.09)	–	–

*STAI-S, State-Trait Anxiety Inventory State-Scale; STAI-T, State-Trait Anxiety Inventory Trait-Scale; BDI, Beck Depression Inventory; FEE, Fragebogen zur Erfassung der Ekelsensitivität, engl. “Disgust Sensitivity Questionnaire”; BVSF, Blut-Verletzungs-Spritzenangst-Fragebogen, engl. “Blood-Injury-Injection Fear Questionnaire”; MQ, Mutilation Questionnaire; MBPI, Multidimensional Blood/Injury Phobia Inventory; BAT, Behavioral Approach Test; GSR, Global Success Rating; tot, total; ^∗^*p* < 0.05; ^∗∗^*p* < 0.01; ^∗∗∗^*p* < 0.001. ^*a*^Effect-sizes not reported if ANOVA-result was not significant. ^*b*^Analysis only includes the first 6 steps. ^*c*^Analysis also includes the 7th and 8th step of the post-BAT. ^*d*^Mean rating at highest step reached*.

At pre-treatment assessment, 80% (*n* = 32) of the participants displayed subjective fear levels equaling or exceeding the cut-off score of 67 in the BVSF. At post-treatment assessment, only 50% (*n* = 20) still did so (χ^2^(1) = 7.91, *p* = 0.005). In contrast, four participants showed an increase in the BVSF score (*M* = 6.5, *SD* = 5.12). Concerning the MBPI, 90% (*n* = 36) of the participants initially displayed fear levels that equaled or exceeded the cut-off score of 48. At post-treatment assessment, 60% (*n* = 24) of the participants still did so (χ^2^(1) = 9.60, *p* = 0.02) whereas only one participant displayed a slightly (one point) higher total score after treatment. Prior to conducting the BAT, 55% of the participants (*n* = 22) reported a history of fainting during blood drawings. At post BAT, 70% (*n* = 28) of the participants were able to undergo a blood drawing procedure (among them 15 individuals with a history of fainting). Eighteen participants (45% of total) were able to observe the procedure closely. Compared to pre-treatment assessment two participants (5%) showed less behavioral approach (-1 step) at post-treatment BAT.

### Are the Effects of LG-OST Stable Over Time?

Twenty-five LG-OST-participants (62.5% of initial treated sample) were available for FU-measures. Mean time between post- and FU-measure was 7.4 months (*SD* = 2.96). To investigate selective drop-out effects, we compared gender with χ^2^-test and all baseline measures for FU-completers and non-completers (*n* = 15) with univariate ANOVAs. FU-completers did not differ from non-completers with regard to age, gender, or years of education (all *p’*s > 0.05). The same was true for the applied general clinical state measures, that is, trait anxiety (STAI-Trait), depressiveness (BDI), disgust sensitivity (FEE) as well as for state-fear levels (STAI-State), all *p’*s>0.05. However, in terms of BII-fear, FU-completers expressed significantly higher pre-treatment fear levels in two of three of the applied subjective fear measures, that is, the MQ, *F*(1,38) = 5.39, *p* = 0.026 and BVSF, *F*(1,38) = 7.82, *p* = 0.008. However, we observed no significant differences neither in the MBPI (*p* = 0.191), nor regarding the maximum step reached in the pre-treatment BAT (*p* = 0.642) or BAT SUD-fear (*p* = 0.312) and disgust ratings (*p* = 0.213). Moreover, we compared pre- to post-treatment differences and percentage treatment gains between completers and non-completers. The latter was required as there were pre-treatment differences. However, neither concerning any pre- to post-treatment difference (all *p’*s>0.27) nor concerning the mean percentage fear reduction (completers: *M* = 19.5%, *SD* = 15.1%; non-completers: *M* = 24.0%, *SD* = 22.4%), *F*(1,38) = 0.61, *p* = 0.44 there were any significant differences. In sum, these results suggest that there has been no sample selection due to the level of immediate treatment outcome and that the below reported LG-OST FU-effects may depict expectable LG-OST long-term results in individuals who rather are strongly afflicted by BII-fear symptoms.

As can be seen in **Table 2**, we observed strong to very strong treatment effects at follow-up in all measures focusing subjective BII-fear. Effect size was weakest for the “*Hospital*” subscale of the MBPI (Cohens’ *d* = 0.81) and strongest for the BVSF “*Injection*” subscale (*d* = 1.87). Our post to FU analyses demonstrate further decreases in BII-fear in most measures within the post-FU interval with mostly moderate to large effect sizes. In contrast, participants’ global rating of treatment success (GSR) decreased significantly from post to follow-up, see **Table 2**.

**Table 2:** Means, SDs, CI’s, and effect strengths (Cohen’s d) of post- to FU changes of blood-injury-injection fear measures for LG-OST-participants who completed FU-measures (*n* = 25).

	Pre	Post	FU	Pre vs. FU	ES [95% CI] Pre vs. FU	Post vs. FU	ES [95% CI] Post vs. FU
	*M (SD)*	*M (SD)*	*M (SD)*	*F*^a^	*Cohens’ d*	*F*	*Cohens’ d*
MBPI tot	92.24 (29.53)	69.84 (32.22)	48.84 (23.81)	55.38	1.62 [0.98 to 2.26]	11.23**	0.74 [0.17 to 1.31]
*Injection*	24.28 (6.90)	17.72 (8.64)	13.28 (7.48)	72.89	1.53 [0.90 to 2.16]	7.45	0.55 [0.02 to 1.11]
*Fainting*	12.38 (7.73)	10.04 (8.21)	6.08 (5.39)	24.20	0.95 [0.35 to 1.54]	5.69*	0.57 [0.01 to 1.14]
*Blood others*	22.04 (8.08)	17.04 (8.06)	12.60 (7.57)	22.00	1.21 [0.60 to 1.81]	7.38*	0.57 [0.01 to 1.13]
*Hospitals*	10.88 (9.28)	7.88 (7.58)	4.72 (5.57)	20.90	0.81 [0.23 to 1.38]	12.10**	0.48 [0.09 to 1.04]
*Blood own*	10.00 (4.81)	7.92 (4.66)	5.36 (3.51)	49.73	1.10 [0.51 to 1.70]	12.76**	0.62 [0.05 to 1.19]
*Injuries*	10.08 (4.00)	7.48 (4.38)	5.68 (3.93)	38.98	1.11 [0.51 to 1.71]	5.59**	0.43 [-0.13 to 0.99]
BVSF tot	95.68 (18.89)	72.64 (19.76)	66.84 (17.29)	55.85	1.59 [0.96 to 2.23]	3.20^#^	0.31 [-0.25 to 0.87]
*BI-self*	22.36 (7.70)	18.08 (7.12)	16.02 (6.33)	27.40	0.90 [0.32 to 1.48]	6.28*	0.31 [-0.25 to 0.86]
*BI-others*	22.56 (5.63)	17.60 (5.58)	16.68 (5.16)	38.51	1.09 [0.50 to 1.68]	0.88	0.17 [-0.38 to 0.73]
*Injection*	22.84 (3.39)	16.00 (5.06)	14.88 (4.96)	92.51	1.87 [1.21 to 2.54]	1.55	0.22 [-0.33 to 0.78]
*Mutilation*	27.92 (8.37)	20.96 (7.06)	19.16 (6.31)	34.40	1.18 [0.58 to 1.78]	2.10	0.27 [-0.29 to 0.83]
MQ	20.35 (4.72)	18.40 (5.46)	14.60 (4.91)	26.63	1.19 [0.59 to 1.80]	13.61**	0.77 [0.16 to 1.30]
GSR	–	5.55 (1.28)	4.81 (0.87)	–	–	10.96**	–0.68 [–1.25 to (–0.11)]

*BVSF, Blut-Verletzungs-Spritzenangst-Fragebogen, engl. “Blood-Injury-Injection Anxiety Questionnaire”; MQ, Mutilation Questionnaire; MBPI, Multidimensional Blood/Injury Phobia Inventory; GSR, Global Success Rating; tot, total. ^*a*^All F-scores were highly significant (*p* < 0.001); ^∗^*p* < 0.05; ^∗∗^*p* < 0.01; ^∗∗∗^*p* < 0.001*.

At pre-treatment assessment, 4% (*n* = 1) of FU-completers displayed subjective fear levels below the cut-off score of the BVSF. This score increased to 24% (*n* = 6) at post-treatment assessment. At follow-up, 52% (*n* = 13) of the participants expressed fear levels below the BVSF cut-off score [pre-treatment to follow-up change: (χ^2^(1) = 16.00, *p <* 0.001)]. We observed comparable results in the BVSF. At pre-treatment assessment, the BVSF-score of 12% (*n* = 3) fell below the cut-off score. At post-assessment this was the case in 36% (*n* = 9) and at follow-up in 40% (*n* = 10) of the participants [pre- to follow-up change: (χ^2^(1) = 6.01, *p* = 0.014)].

At follow-up assessment, 44% (*n* = 11) of the participants reported that they actively sought out blood- and injection relevant situations encouraged by the treatment within the post follow-up interval, and 52% (*n* = 13) of the participants reported to feel “safer” or “much safer” in dealing with BII-relevant situations.

### What Are Predictors for LG-OST-Outcome?

To identify potential outcome predictors, we initially calculated pre to post difference scores of the MBPI, BVSF, MQ, and the BAT (steps and associated SUD-ratings). None of these change scores were significantly correlated with age, years of education, clinical baseline-status (BDI, STAI-Trait), or disgust sensitivity (FEE), all *p’*s > 0.10. Only within the BVSF, we observed a positive correlation between the level of fear reduction and the BVSF pre-treatment score (*r* = 0.47, *p* = 0.002). This was not the case for the MQ and MBPI. The increase in approach behavior at post-treatment was significantly associated with pre-treatment state-fear level (*r* = -0.42, *p* = 0.006).

## Discussion

This Phase I study aimed to investigate feasibility and effectiveness of a large-group one-session fear treatment (LG-OST) in a sample of 40 individuals highly fearful of blood, injuries and injections. Prior LG-OST trials conducted in spider fearful (Wannemueller et al., 2016) and dental fearful individuals (Wannemueller et al., 2017) were already promising, and the results of the present study clearly underline the feasibility and usefulness of LG-OST protocols.

In terms of mere feasibility, our initial concern regarding patients’ doubts about the effectiveness of the treatment and a possibly consequent weak participant-inflow proved ungrounded. When initially conceptualizing the LG-OST intervention, we also were concerned about potential mass panic or aggravation during the trial. None of these concerns proved true in the previous dental fear or spider fear LG-OST conditions. However, eight participants displayed heavy pre-syncopal symptoms or actually fainted during the present trial, all while watching the expert-video presented for psychoeducative purposes. They were quickly stabilized by medical professionals present during the entire trial, and all of them were capable to complete the treatment. First, this experience emphasizes the necessity of having medical professionals present during future LG-OST trials. Second, due to this experience, we recommend either to design the imparting of psychoeducative information less pictorial or to refrain completely from the use of psychoeducative material containing detailed descriptions of BII-relevant procedures. Such material may evoke heavy vasovagal symptoms triggered by unwanted exposure to fear-relevant contents participants are not prepared for at this stage of the intervention. In accordance with this assumption, when participants were prepared in advance for planned exposure exercises by learning and applying bodily coping strategies, all participants were capable to control emerging signs of upcoming vasovagal response.

Regardless of the mentioned issues concerning the conceptualization of a LG-OST targeting BII-fear, the protocol applied here led to substantial short- and long-term subjective BII-reduction and, to a lesser extent, also to a change in avoidance behavior after treatment and associated feelings of disgust (BAT and SUD ratings). We observed post-treatment reductions of subjective BII-fear levels on all questionnaires with effect sizes ranging from medium (MQ) to large (BVSF, MBPI). The exploratory correlation analyses suggest LG-OST to be effective for a wide range of individuals, as improvement was not associated with sociodemographic state variables, relevant traits (BDI, trait-fear, disgust sensitivity) or pre-treatment fear levels, except for the BVSF. Analyses of the MBPI- and BVSF-subscales showed that our LG-OST protocol was particularly effective with regard to injection fear. This assumption is supported by the behavioral data: 70% of participants were able to undergo a blood drawing after being treated and reported less fear and disgust during the procedure. Most of these individuals (64% of the participants who underwent a blood drawing) were even capable to closely observe the procedure and to control possible emerging vasovagal responses by using the learned coping strategies. Given that the subjective fear levels at pre-treatment assessment resemble those reported in phobic cohorts, this is a quite remarkable finding.

Considering treatment content, it is little surprising that the observed change in the MQ was relatively small given that all of our live exposure and video exposure exercises consisted of blood drawings. Exposure to wounds, injuries and mutilations only took place during our pictorial exposure exercises. Also Öst et al. (1992), who introduced one- and five-session protocols that comparably to ours primarily consisted of various injection-procedures (albeit all administered as *in vivo* exposure exercises), reported only moderate post-treatment changes in the MQ. However, the pre- to follow-up change in the MQ (*d* = 1.17) for our LG-OST resembles the follow-up effect sizes reported for individual OSTs, regardless whether the OSTs were exposure-based (*d* = 0.90, Öst et al., 1992), used applied tension (*d* = 1.13, Hellström et al., 1996), or tension only (*d* = 1.15, Hellström et al., 1996). Unfortunately, besides MQ, the individual OSTs used subjective fear measures different from ours. Therefore, we cannot directly compare the reported BVSF or MBPI effects with effects reported for individually treated samples.

Interestingly and in contrast to the observations in previous LG-OST trials, we found substantial fear decreases within the post-FU interval in individuals who returned for the FU-assessment. Consequently, there were large to very large subjective pre- to follow-up fear reductions in all measures, by far exceeding those reported in previous LG-OST trials.

One can only speculate why the LG-OST targeting BII-fear might have especially stimulated between-session improvement. One reason could be that new learned coping strategies helped participants to control their bodily symptoms, which in turn enabled them to handle and to confront themselves with BII-relevant situations during the post-FU interval. To further support this argument, about half of the follow-up completers stated that, encouraged by the treatment, they actively approached BII-relevant situations during the post-FU interval. Moreover, it is possible that compared to spider- or dental-fear, in BII-fearful individuals fear-evoking expectancies are quite homogeneous. In turn, a highly standardized LG-OST might target fear-evoking expectancies especially well and promotes the acquisition of new non-threat expectancies for BII-associated situations (see Craske, 2015 for a detailed explanation of factors leading to successful between session exposure). However, future research focusing on the mechanisms underlying treatment outcome in SPs should investigate whether treatment outcomes vary with respect to how homogeneous fear-evoking expectancies are in the suffering individuals and, thus, how they can be best targeted by highly standardized treatment formats such as the LG-OST.

Our study exhibits several limitations. As customary for Phase I trials, we did not include an untreated or placebo-treatment control group in this feasibility-trial, which could compromise the validity of the findings, for example the effects of repeated measurement or regression toward the mean. Furthermore, the number of LG-OST completers who returned for FU-assessment (62.5% in total) was relatively low, and our analyses yielded that drop-out was not random since completers expressed higher pre-treatment fear levels compared to non-completers. However, this finding suggests that our follow-up results are in line with results in clinical samples consisting of individuals strongly affected by BII-fear symptoms. The relatively low response rate at follow-up assessment might be related to our attempt to minimize participants’ doubts to participate in a large-group training by guaranteeing anonymity. Therefore, in turn, participants were required to actively waive anonymity in order to be contacted and scheduled for a follow-up appointment. Moreover, identification of outcome-moderators was restricted as the examined sample, e.g., in terms of demographic variables was highly homogeneous and as sample size in general was relatively small. Due to ethical reasons, we were not allowed to draw blood twice on the same day. This clearly restricts the value of our behavioral assessments since pre- and post-tests did not include the same number of steps and thus were hardly comparable.

In spite of the weaknesses mentioned, we conclude that a LG-OST targeting BII-fear represents a very valuable treatment tool, both as a single-treatment and as an intermediate step within a framework of stepped care of phobic fear. Prior experience with LG-OSTs in spider-fear and dental-fear (Wannemueller et al., 2016, 2017) suggests that LG-OST effects are inferior to individual OSTs, which led us to the conclusion that LG-OST protocols might sufficiently address the needs of fear treatment of many but not all participants. However, the effects of the present LG-OST-trial in BII-fearful individuals equaled those reported for individual OST formats (see Ayala et al., 2009). Thus, in medical settings, a LG-OST targeting BII-fear might serve as a single treatment option to prepare fearful individuals for BII-relevant procedures.

Moreover, given their very high efficiency, LG-OST approaches might represent a useful step within a stepped care model of SP treatment. Within this framework, patients could first be referred to very low intensive treatments, such as bibliotherapy or self-help books. Those in need of additional treatment could then progress to LG-OSTs or web-based group treatments as the next, moderately intensive treatment option, while only LG-OST non-responders would finally progress to individual, multi-session treatment formats.

Besides clinical usefulness, applicability of very short, highly standardized group treatment formats such as LG-OST may offer novel research opportunities. Using feasible and effective LG-OST-protocols allows researchers to investigate moderators and mediators of treatment success under highly standardized environmental conditions, as well as enables an easy transfer of the mechanistic findings from lab-based research into a more natural fear-related context.

## Conclusion

A large-group one-session treatment (LG-OST) combining exposure with applied tension or relaxed breathing proved feasible and very effective with regard to fear of blood, injury and injections. At follow-up assessment, we observed large to very large reductions of subjective fear levels equaling those of previously reported individual OSTs as well as a decrease in behavioral avoidance. Due to their high efficiency, LG-OSTs may prove to be valuable treatment tools in patients with phobia, for example within a stepped care approach. A LG-OST protocol targeting BII fear could also be considered as a single treatment option due to its very high effectiveness for instance when fearful patients are prepared for BII-relevant procedures in medical settings.

## Ethics Statement

The study protocol was approved by the local ethics commitee of the faculty of psychology at Ruhr-Universität Bochum. All subjects gave written informed consent in accordance with the Declaration of Helsinki.

## Author Contributions

AW, JM, AF, ZK, and KW conceived and designed the study. AW analyzed the data. AW, JV, and SS wrote the paper.

## Conflict of Interest Statement

The authors declare that the research was conducted in the absence of any commercial or financial relationships that could be construed as a potential conflict of interest.
